# Foreign

**Published:** 1841-12-11

**Authors:** 


					﻿FOREIGN.
The following forthcoming volume cannot
fail to add essentially to our stock of informa-
tion from the rich graneries of London. It is
a symptom of the advance of science in that
Capitol that comparative anatomy and anato-
mical monographs of rare animals should be
included in the catalogue of intended contents;
for there is scarcely any department of medi-
cal science which may not be continually im-
proved by that kind of generalization which
results from observing extensively the anato-
mical and physiological peculiarities ofbeings
inferior to man. The conclusions drawn from
the examinations of a few facts or a few ge-
nera only, are often productive of false theo-
ries, which, in their application, lead to most
unfortunate results. A series of experiments is
in progress in France, and has been for years,
with a view to ascertain how far gelatine is capa-
ble of maintaining life; and it would appear
that, when exclusively employed, weakness
and death are rapidly induced! The question
bears upon the supply of food to the whole
French army and marine. We have avoided
commenting upon these experiments as they
are reported, because they have been chiefly
confined to a few animals, mostly of the car-
nivorous class; and, being well aware that
goats, or sometimes sheep, will eat tobacco with
avidity—that some of the former relish white
lead as a condiment—that a little ipecacuanha
is said to kill a dog though he takes large doses
of arsenic almost with impunity, we prefer
awaiting the ultimate observations of the learn-
ed observers, before adopting any conclusions
in relation to their hygienic and economical
value. But the great importance of such re-
searches, when sufficiently elaborated, is too
obvious to need a comment.
Proposed Transactions by the Royal College
of Surgeons in London.—The Council propos-
ing to publish, in the course of the ensuing
year, a volume, to be entitled “Transactions of
the Royal College of Surgeons in London,”'
invite, from the members of the College, and
other scientific persons, communications relat-
ing to the improvement of anatomical and sur-
gical science.
The subjects proposed to be included in this
publication are specified in the following ex-
tract from the ordinances of the College?_
“The Transactions shall consist of Original
Communications on Surgical subjects : &Col-
legial and Jacksonian Prize Dissertations,
deemed of sufficient originality and merit:
Original Memoirs on Human Anatomy ; Ori-
ginal Memoirs on Comparative Anatomy;
Anatomical Monographs of rare Animals, dis-
sected in the Museum of the College: Expla-
nations of, and Commentaries on, important
Preparations in the Museum, with illustrative
Plates; Statistical Reports from Hospitals. It
is requested that papers intended for publica-
tion in this volume may be transmitted to the
President, at the College, on or before the 1st
of May, 1842.
Edmund Belfour, Secretary,
July28lh,lMl.
Ed. Med. and Surg, Journ.
Physiological and Pathological remarks on
the circulation in the brain.—We rarely ven-
ture to quote largely, without condensation,
from the clinical lectures published in the
London Journals. Many of these, being de-
signed for students just entering upon their
career, are scarcely worthy of record in pages
designed for permanent usefulness, and others
form portions of long series, and could only be
presented, with justice, in the form of volumes.
From both classes we obtain, occasionally, a
few pertinent observations fitted for our peri-
scope; but the following selection, which
comprises about two-thirds of a lecture at St.
George’s Hospital, is so beautiful in model and
important in matter, that it could not be con-
densed with justice either to the author or
the reader.
“ Affections of the head are al ways an inter-
esting subject of consideration, and there are
several at the present time under your notice,
of which I will select three, as they offer some
important practical suggestions; and some
questions have been asked me, which show
that several points connected with them are not
well understood by all of you.
I.	The first case is that of a little boy, Wil-
liam Lyons, eetat. 13, admitted March 24th, into
Cholmondely Ward, with caries of the anteri-
or part of the right parietal bone, with an open-
ing through the skin,’ about the size of a six-
pence, exposing the dura mater. Tongue
clean; pulse quiet; no pain in the head;
sleeps well at night; countenance pale. States
that about three months ago he was carrying
some chairs on his head, when he happened to
fall down, and received a blow on the head
from one of the chairs. He was not insensi-
ble after the accident, but only perceived a
swelling on the head after the blow. This
did not disappear, and was opened about a
month afterwards, when some pus escaped,
since which time it has continued to discharge,
but he has not perceived any bone come away.
This account is corroborated by the informa-
tion we have received from the House Sur-
geon of the Westminster Hospital, where he
was admitted a fortnight after the accident,
and where the swelling was punctured, and
found to contain only pure pus; but this gen-
tleman says that bone was felt deeply situated,
as if in the substance of the brain. During
all the time he was there he suffered from no
symptoms of consequence, and neither brain
nor bone escaped through the opening. At the
time of his admission our notes say the probe
passed under the scalp to some distance be-
yond the opening, (about an inch and a quar-
ter by an inch, in size ;) and bone is felt of ap-
parently the natural thickness, with an abrupt
margin on each side: In the interval no bone
is felt, and only the dura mater (as it seems)
presents itself, which is vascular, and bleeds
from the touch. The pulsation of the brain is
also seen in the whole space where the bone
is deficient, as if there was at present no de-
pressed bone in that situation below its natural
level.
1.	Such is the history which was obtained
of this boy when he was admitted, and from it
we should gather that the bone had been
broken and depressed at the time of the acci-
dent, with laceration of the dura mater, fol-
lowed by slow suppuration: ifitwereso, there
is no account of any bone having ever come
away, and therefore it must still remain in,
since the surface exposed is at too great a
depth to make us suppose that the depressed
piece could have remained partially attached to
the dura mater; and unless so attached, it, of
course, cannot have been absorbed, as the pro-
cess of absorption of bone requires some living
vascular action. If the bone is lodged in the
brain, it has, at any rate, excited not the least
irritation since the boy has been in the hospi-
tal, and it appears that he has not suffered pre-
viously from any affection of the brain. I must
confess, however, that I have some doubts up-
on this point, since the living surface, which
is visible, is quite smooth and level, and looks
just as the dura mater would have done if it
had been uninjured. What can have become
of the bone then, you will ask 1 Why, it is
not at all impossible that the injury, and the
pressure of blood effused under the pericrani-
um, may have caused the absorption of a por-
tion of bone with caries, which would go on
enlarging the opening when once formed,
especially in a scrofulous boy, since you know
that scrofulous caries, taking place spontane-
ously, will occaasionally produce this result.
And the course of the local changes, since he
has been in the hospital, is just like such an
action, as there is some thickening of the soft
parts around the opening, and another spot
lower down has suppurated in the scalp, and
the caries, which now exists, has been attend-
ed, as it often is, by the death of some of the
diseased bone; so that in the early part of
May I removed several small pieces of dead
bone from around the opening, in that part of the
bone which preserves its natural level ; and
some of this may have been felt when the ab-
scess was opened. I will not dwell more,
however, upon this part of the case, but will
pass on to a second event in his history, which
is interesting, though not connected with the
brain.
2.	The boy was observed, on his admission,
to be very languid, and pale, and weak, and in
a few days he had a good deal of cough, with
some pain in the side, and we found that he
scarcely breathed with the left side, which was
quite dull, except just at the upper part; and
in a day or two more we found that this side
was actually larger than the other : in short,
he must have had pleurisy before he came in,
with effusion of water or other liquid into the
chest, sufficient almost entirely to compress
the lung, so as to prevent the ingress of air.
The little irritation he had, when our attention
was first drawn to the chest, was easily sub-
dued, particularly by blistering; andon the
8th of April our notes tell us that more air
seemed to enter the lung, but at the same time
that he had more cough, no doubt from the
compressed lung beginning to expand a little,
as some of the fluid was absorbed. He con-
tinued to have a little cough, but no constitu-
tional irritation; and the fluid was gradually
absorbed, but without much further expansion
of the lung, till, our notes tell us on May 24th,
that no air enters the chest below the fourth
rib under the scapula, and in front it cannot
be heard at all ; but now, instead of the chest
on this side being larger than the other, the
thorax is perfectly flattened, and measures an
inch and a half less from the spine to the cen-
tre of the sternum than on the other side. The
heart at the same time beats over a large
space, and is enlarged, as it would appear,
from rheumatism. You have thus an excel-
lentexample of the absorption of fluid from the
chest without operation, while the lung has
only expanded to a small extent; and the ef-
fect of this alteration of siz& is generally to
make the spine curve to the side, with depres-
sion of the shoulder.
3.	I will, however, chiefly dwell in this
case on the state of circulation in the brain, as
you can see it going on through the aperture
in the skull, as it does not seem to be well un-
derstood among you all; and there are many
important inferences to be derived from a con-
sideration of it, accounting, as this considera-
tion will do, for the obscurity so often ob-
served in injuries and diseases of the brain, in
which exactly the same symptoms can often
be traced to different, or even exactly opposite
causes.
a.	Look, in the first place, at the exposed
surface of the brain in its habitual, I was go-
ing to say in its natural stale, and you will see
that there is an arterial pulse in it, so that there
is an alternate rising and falling exactly cor-
responding with the systole of the heart: it
rises as the arteries beat, and falls again till
the next stroke of the heart; in other words,
the brain actually contains more arterial blood
at one time than at another.
b.	Look again, when I have opened an ab-
scess, or taken out a piece of bone, or when he
has risen quickly off the bed, or has been sub-
jected to any other kind of excitement, and
you will perceive that the cavity below the
aperture is lessened, and that there is also
more pulsation than in the quiet condition;
that the brain is kept permanently higher,
rising and falling alternately, but quicker than
before, and never falling so low ; containing,
in short, a little more blood altogether, and
having more entering it in a given time.
c.	Then make him breathe deeply, or cough,
and you will see that the brain rises still high-
er, so as even to expel the matter in a
stream by the impulse of coughing; in fact,
besides the beating of the pulse, there is a
rising and falling of the surface in correspond-
ence with respiration ; that is, not only 80 or
90 times from the pulse, but 18 or 20 times,
to a greater degree, still from breathing. Dur-
ing each expiration the chest is depressed, so
that the venous blood is prevented from de-
scending, and is accumulated in the veins and
sinuses of the brain, so as to elevate the dura
mater, which again falls in each inspiration
by the weight of the atmosphere, the air enter-
ing the air-vessels, and the blood getting into
the veins of the chest by the elevation of the
ribs and the depression of the diaphragm, out
of the distended veins of the brain.
But most of this is only because there is
an opening in the skull, allowing the air to en-
ter and make pressure on the surface of the
brain, and you are not to suppose that the na-
tural state of the circulation is exactly of the
same kind. When the cranium is entire, the
atmospheric pressure can only be felt at the
various orifices at the base of the skull, and
will there prevent any such vacuum between
the dura mater and the level of the skull as
we can now see; and as the substance of the
brain is incompressible, there cannot possibly
be the variation in the quantity of blood con-
tained in the brain, which there is in our pa-
tient Lyons, with such a deficiency of the
bone.
a.	With regard to the arterial pulse in its
natural state, we see more blood in the arteries
during the systole of the heart, making the
brain rise. So it is also when the bone is
entire ; but as the brain cannot rise, there must
be at that time less in the veins of the brain.
b.	So again in the excited condition of the
circulation, we can see more arterial blood con-
stantly in the brain. There is also, with an
excited pulse, more arterial blood always in
the head, if the cranium is entire ; but then
there must be still less in the veins than in
the quiet state of the circulation, as the brain
cannot rise upwards as we see it now do.
c.	And, with regard to the elevation of the
brain in our patient in expiration, there is also
a similar impediment to the descent of the
venous blood, when the bone has no aperture
in it; but if the veins and sinuses contain more
blood, there must be less in the arteries, which
therefore are not at those times allowed to be
so well filled by the action of the ventricle as
in inspiration.
Thus, theli, in a natural circulation there is
a variation in the relative quantity only of the
arterial and venous blood, not in the absolute
quantity contained in the whole brain ; but as
this variation is taking place every second of
time, you may easily believe that it must be of
great importance to the functions of the brain.
But this is not all: there are other circum-
stances to be taken into account in the physi-
ology and pathology of the brain.
d.	If the brain contain a large relative
quantity of arterial blood, this will be circu-
lating chiefly in the interior of the nervous
substance, separating the fibres and elementary
particles, and stimulating their substance, it
may be, to a healthy performance of its func-
tions ; or it may be, to an undue degree, pro-
ducing inflammation or other mischief.
e.	If, on the other hand, there is a dispro-
portionate quantity of venous blood, this will
be circulating chiefly on the surface of the or-
gan, including the spaces between the masses
of the encephalon ; whence we may under-
stand the effects of venous congestion in the pro-
duction of sleepiness, stupor, paralysis, or other
signs of weakening of the functions of the brain,
or perhaps, like the influence of poison on the
brain, causing delirium, convulsions, apoplexy,
and so on, by means of this black blood.
f.	But, further, we have seen a difference
not in quantity only, or relative proportion of
blood of different qualities, in different periods
of the same kind of circulation, but in the ra-
pidity also with which the same relative
quantity of arterial blood passes through the
brain in a given time. Much more arterial
blood will actually circulate through the brain
with a strong heart than with a feeble one, or
with a quick pulse than with a slow one, the
strength of the heart being the same :—or a
feeble heart, beating very rapidly, may send
more blood through the brain than a stronger
one acting more slowly;—and a continued
steady impulse on the substance of the brain
must be very different in its effects from those
of an irregular or intermittent state of circula-
tion through its texture.
g.	Then, finally, besides all these varieties
of circulation through the whole brain, influ-
encing all the encephalon alike, you must re-
collect that there is irregularity as to particu-
lar parts of these organs. Doubtless, for ex-
ample, there is a greater state of vascularity in
the forepart of the brain in this boy than there
is naturally; and if this be so, when there is
no aperture in the cranium, and the patient
often refers us to one particular spot as the seat
of pain, when there is an abscess or a tumor,
there must necessarily, in accordance with
what I have previously said, be less blood than
natural in the remainder of the brain.
Now all these circumstances influence our
practice in the treatment of cerebral affections
of any kind. We take away blood, and give
nitre or tartar emetic, and use other measures
to lessen the quantity of arterial blood, or mo-
derate the force with which it enters and cir-
culates through the brain, when there is undue
action of the heart, or what is called determi-
nation of blood to the head. We can relieve
the oppressed brain in many cases of plethora,
or venous accumulation, by taking away a
small quantity of blood, so as to enable more
arterial blood to circulate; and thus very often
we restore the functions of the brain and of
the lungs at the same time, whether the cause
of the oppression may have originated in the
brain itself, or in the heart, or in the lungs,
these several organs being so connected to-
gether.
In another case of passive venous conges-
tion we overcome it by stimulating the heart,
so as to make it propel more red blood up-
wards ; or even by change of posture, so as
to facilitate the passage of the venous blood
downwards: the difference of lying down or
sitting up being sufficient to determine a
question of life or death. Then, again, we
can give stimulants to a feeble heart so as to
restore a healthy action to the brain, when in-
sufficiently supplied with arterial blood,
whether in absolute quantity, or in rapidity, or
force of propulsion. You can any day seethe
influence of this latter circumstance in cases of
delirium trauinaticum, in which perhaps the
pulse may be beating at the rate of 150 in a
minute, with great apparent quantity of blood
in the vessels of the head ; but where your pa-
tientwill yet die with furious delirium, unless
you increase the force of the arterial impulse,
by some of that gin by which his brain has
been habitually stimulated, and by which he
will directly be quieted of his violent exer-
tions of mind and body, and fall perhaps into
a gentle and refreshing sleep. Or, again, if
we see a person dying from loss of blood, and
insensible because the brain has so little sti-
mulus of its necessary circulation, we may
perhaps, when time is not afforded for food or
drink to enter the blood, restore his conscious-
ness and save his life, by transferring some
from another person, which may directly be
sent up to our patient’s sensorium, to afford it
the requisite and natural stimulus.
II. No wonder, then, that we should be
offered some great similarity of symptoms
from apparently opposite causes; because, in
fact, these opposite causes may, in effect, pro-
duce the same state of circulation in the brain,
in one of the several circumstances which we
have been considering. No wonder, moreover,
that we should often be at a loss in our diag-
nosis of these causes, and actually puzzled
whether we should bleed or stimulate our pa-
tient—whether we should increase or lessen
the vigour of his circulation. To illustrate
this point, let me next draw your attention to
another case now in the hospital; it is that of
Jane Looker, aet. 32, admitted May 24th, into
Wellington Ward, with these symptoms:—
Complains of great pain in her head, equally
diffused over the whole of it; pulse 90, regu-
lar, and rather weak; tongue clean and moist;
bowels open; no anxiety of countenance, or
frowning; not much appetite. She states that
about a month ago she received a blow on the
upper part of the head, which stunned her for
about ten minutes, ever since which time she
has had great pain in the head. She was cup-
ped a week since, which relieved her for a
time, but, owing to working hard and drinking
some porter, the headache returned, and is now
as bad as ever. She is at present suckling a
child eight months old, and has been living-
poorly of late, and was getting thin and out of
health before the blow.
Here, then, is a case that might at first be
regarded as an instance of inflammation after
concussion, from the acuteness of the pain the
woman complained of. You find that this
view was taken of the case before her admis-
sion, and she was actually relieved by the cup-
ping which was ordered. So it is, however,
in many cases of weak and irregular circula-
tion, and the temporary relief is succeeded by
an aggravation of the pain. You find again
that she attributes her return of pain to the
stimulus of porter; but neither does this prove
that the stimulus was injurious, since she was
at the same time imprudent enough to return to
hard work, which would naturally make a weak
person suffer.
The whole state of the patient, however, left
me little room to doubt that the pain was not
likely to be inflammatory, and particularly the
colour of the countenance, and the absence of
anxiety and frowning, which inflammation al-
most always produces; the absence of sharp-
ness and force of the pulse, although it was
somewhat quick and jerking, and then the dis-
covery of the fact of her having suckled up to
the present time, and that in a condition of
great privation. This long-continued suckling
is, without any injury, a fertile source of de-
rangement of the health generally, and of amau-
rosis, pain in the head, or mania in connexion
with the brain.
I let her lie quiet, then, with some cold lo-
tion on the forehead, till the day after her ad-
mission, giving her only some more nourish-
ment. The pulse the next day, the 25th, was
only 80, and soft; and the pain was a little
better, but still much complained of. I there-
fore ordered her some ammonia draughts, and
a small blister to the nape of the neck. On
the 29th, our notes say the pain was rather
better.
On June 1st 1 gave her some bark and aro-
matic confections, the countenance being more
cheerful and a little more florid, but the pulse
still weak, and gave her also a little porter.
On the 5th it seemed as if this was rather
too much, the pain in the head being still com-
plained of as much as before; and I gave her
some Sp. Ammoniae Fcelidus and Camphor
Mixture instead of the bark, letting her diet re-
main as good as it had been; and now you may
see her convalescent.
There are many such cases as this which 1
will come under your notice after injuries of i
the head, in which, if you are in any doubt,
you should do little, but rather watch your pa-
tient; or, if your opinion is a little inclined to
either side, you should proceed very cautious-
ly, whether with the plan of depletion or of
stimulation, lest you should find yourselves in
error, or lest a change of plan should proceed
a little too far; and there are many cases in
which you will require even a combination of
both principles of action. A man, for instance,
fell from a ladder, and was bled after his ad-
mission for the symptoms of concussion, the
house surgeon not being aware that he had also
been bled just before his admission and very
soon after the fall: he was for many days in a
state of complete oppression of brain, with a
slow and laboured pulse, and nearly total in-
sensibility, and from want of nervous power
there was the same torpor of bowels which is
so much more cojnmon in diseases than in in-
juries of the brain. Now the state of this man
was much more owing to large loss of blood
than to the fall, and his tongue was thickly
coated with a white cream that indicated a very
disturbed state of circulation in the brain, but
is not usual as a consequence of injury; but
although this man required a good deal of light
nourishment, and some diffusible stimulants
to hasten the passage of the blood through the
brain, he was also much benefited by blisters
to the neck, and by small doses of calomel and
opium.
In some of these cases you will find even a
cupping glass, or a few leephes to the forehead
or temples, when there is intense pain, not at
all incompatible with moderate stimulation of
the general system ; local congestion or inflam-
mation being actually the consequence of fee-
bleness of the circulation, and requiring the
same local remedies, and the same effects on
the capillaries of the part, which would be re-
quisite in another case in which the general
circulation was in an opposite condition; and
for this purpose a blister, which I gave to our
present patient, is very efficacious,—London
Med. Gaz.
Every thing connected with the physiology
of the hip-joint that wears the air of novelty,
derives importance from the obscurity of the
diagnosis of many affections of that deeply
seated articulation, and we therefore make no
apology for the extraction of the following
paper entire, though it is somewhat prolix in
style.
On the Uses of the Round Ligament of the
Hip-Joint. By James Luke, Esq., Surgeon to
the London Hospital.—Amongst the contribu-
tions to anatomy and physiology communicated
at various times to the Anatomical and Physio-
logical Society in Edinburgh, by Dr. Knox, is
one on the round ligament of the thigh-bone
in man, published in the July number of the
Edinburgh Medical and Surgical Journal, in
which, after stating that ever since he taught
anatomy, he has been much struck with the
contradictory statements in respect to the uses
of the round ligament, he proceeds to set forth
the opinions of many writers of high authority
upon the subject, as of Dr. Barclay, M. Boyer,
M. Meckel, Sir A. Cooper, M. Cruveilheir,
M. Gerdy, M. Blandin, and Mr. Mayo, and
finally concludes with the expression of his
own; in which he in some measure differs from
the individuals just named. These opinions
are, for the most part, to the following effect:
1st. That it restores the head of the femur to
a right direction when it has partially quitted
the cavity of the acetabulum. 2d. That it
prevents the femur from leaving the cotyloid
cavity upwards and outwards. 3d. That it
opposes luxations upwards, outwards, and
downwards. 4th. That it has a tendency to
prevent dislocation in all directions, particu-
larly downwards. 5th. That it limits rotation
inwards of the femur. 6th. That “ rotation,
which is either outwards or inwards, can be
carried only to the extent allowed by the round
ligament of the femur. All attempts, there-
fore, to combine to any great extent the ele-
ments of rotation with other movements of
which the joint is capable, receive a complete
check from the presence of the round liga-
ment, and more especially the motion of ad-
duction, which cannot be performed at all when
the limb is at the same time rotated outwards
or inwards.”
“Flexion inwardsand outwards, combined
with rotation, is also in an especial manner
checked by the presence of the round liga-
ment.”
This latter is the opinion held by Dr. Knox
himself, in support of which several experi-
ments were made by him, confirmatory of his
views. Although it was found in one of these,
that, after the round ligament was divided,
“ the capsular ligament checked the motions of
the femur just as effectually as when the round
ligament was left intact,” it is not intended to
question here their accuracy, or the deductions
which have been drawn from them.
It will be the business of the present com-
munication to set forth other uses to which the
round ligament is destined, of infinitely greater
importance in the economy of the articulation
than any which have been assigned to it by
any of the authorities above mentioned. These
uses are of such a character, that, in my esti-
mation, they well deserve the appellation of
primary, while those which have been just
enumerated, should be considered as merely
subordinate and secondary; and the whole to
afford another illustration, in addition to the
many which may be found in the body, of the
adaptation of the simplest means to the attain-
ment of most complex results.
On a general survey of the articulating sys-
tem, it will not fail to strike the observer, that
each joint which enjoys motion to any extent,
is furnished with the requisite facilities to that
end; but if the attention be particularly di-
rected to those joints upon which the weight
of the superincumbent body falls when in the
erect position, such as the joints of the foot,
knee, and vertebral column; the fact of there
being in these an additional provision, whereby
the violent shocks received by one part are miti-
gated, or altogether prevented from being com-
municated to another, will not fail to be as
readily observed;—and the springing arch of
the foot, the interarticular cartilages of the
knee, and the intervertebral substance, will be
recognized as so many stops to the concussion
of the body during the acts of walking, run-
ning, jumping, and other exercises.
Although the omission of the hip-joint from
the catalogue of those provided with stops to
concussion has been made, yet it will be found
in the sequel, that the round ligament, by its
arrangements, and by its attachments, is fully
equal to prevent the forcible contact of the
head of the femur with the upper part of the
acetabulum during the motions of the joint;
from which circumstance several results follow
besides that of the stoppage of concussion, as
will be subsequently enumerated. In the first
place, it will be well to determine, the truth of
the observation, that the head of the femur and
the acetabulum do not come into forcible con-
tact while the round ligament is entire and in
a normal condition. With this view, proba-
bly the relation of two experiments made upon
the hip-joints of a man who died in the Lon-
don Hospital from fracture of the skull, will
be the most satisfactory course to be pursued.
The first experiment was performed in the
following manner on the left hip : After the re-
moval of the muscles from the anterior and
lateral parts of the capsular ligament, as well
as from the venter and dorsum of the ilium, a
cut was made with a saw, beginning from the
anterior inferior spinous process, and extending
through the brim of the pelvis : another cut was
made also through 1he brim of the pelvis, but
extending from the outer extremity of the ramus
of the pubes. These two cuts were connected
by a third, made nearly parallel with, butdeeper
in the pelvis than its brim. After which, the
bone, insulated by the saw, was raised by the
chisel. By this proceeding the whole upper
part of the acetabulum was removed, while
the anterior superior spinous process remained
entire, and furnished a point from which mea-
surements of the thigh could be made. As
there was not any obstacle from bone remain-
ing to the ascent of the femur into the abdo-
men, by reason of the removal of the upper
part of the acetabulum, an opportunity was af-
forded of determininghow far that ascent might
be prevented by the round ligament, or by
other causes. Attempts were therefore made
to forcibly push up the whole limb towards the
pelvis; but the attempts merely produced an
increase in the tension of the round ligament,
without any shortening of the distance between
the superior spinous process and the patella, as
ascertained by most careful measurement and
comparison with the opposite side. Having
determined that there was not any shortening
of the thigh, (the round ligament being entire,)
a scalpel was introduced, and the ligament di-
vided completely through, without any further
division of the structures surrounding the joint.
A considerable shortening of the thigh now
was affected, by pushing the limb towards the
pelvis; nor did there appear any obstacle to its
ascent until the head came in contact with the
ilium, at the uppermost point at which it had
been sawn through.
Although this experiment was performed
with every care that the measurement should
be accurate, it is possible that a very trifling
amount of shortening, such as would be suffi-
cient to bring the head of the femur and upper
surface of the acetabulum in forcible contact,
might have been passed over unnoticed.
The second experiment was therefore made
on the opposite hip. The capsular ligament
being bared as in the former experiment, an
incision was made perpendicularly through its
centre on its anterior surface, after which, the
outer and upper part of the acetabulum was
removed by two saw cuts, one of which was
continuous with the incision of the capsular
ligament; the other traversed from the anterior
inferior spinous process to the upper termina-
tion of the first. Thus about one-half of the
upper part of the joint was exposed, and an op-
portunity afforded of examining, at the edge
of the perpendicular saw cut, to what extent
the acetabulum was pressed on by the head of
the femur when the limb was pushed towards
the pelvis by an assistant. When the limb
was so treated, it was observed, that the op-
posing cartilaginous surfaces of the upper part
of the acetabulum, and of the head of the fe-
mur, were brought barely in contact with each
other, and that the thin point of a scalpel intro-
duced between them was held by no means
tightly. A different state of things was induced
immediately that the round ligament was di-
vided. The same surfaces were now brought
forcibly into contact by pressure on the foot,
and a scalpel, introduced in a similar manner
to that just mentioned, was held with a degree
of tightness very far greater than when pre-
viously introduced. The force of contact be-
tween the head of the femur and the acetabu-
lum was regulated, therefore, by the round
ligament, which, when present, prevented more
than mere contact taking place.
The inferences to be drawn from these expe-
riments necessarily induce the supposition, that
other and more important uses than those as-
signed to it at the commencement of this paper
belong to the round ligament : while the expe-
riments themselves directly point out the na-
ture and extent of those uses.
It must be obvious that two surfaces, pre-
vented from forcible contact with each other,
cannot transmit easily from one to the other
any concussion that either might have received.
It is equally obvious that, by the same preven-
tive, friction and its effects are diminished, if
not altogether removed from the surfaces,
whereby the motions become less encumbered,
and acquire a freedom which they would not
otherwise possess. It also follows from the
premises, that, in the act of standing, the round
ligaments of the opposite side serve as cords of
suspension for the trunk on the heads of the
thigh bones. For assuming the pelvis, to
which the round ligament is attached by one
extremity, to be the fixed point, it has been
shown that the thigh is prevented by it from
ascending or from becoming shorter. But if
the thigh itself be assumed to be the fixed
point, to which the ligament is attached by its
other extremity, the trunk, then beooming the
moveable body, has a tendency to descend,
which tendency is counteracted by the liga-
ment, by means of which the trunk is suspend-
ed from the thigh or fixed point. The body,
therefore, while erect, iri reality swings upon
the two round, or, as I would call them, sus-
pensory ligaments of the hip-joints ; but stea-
died in that position by the enormous muscles
passing from the pelvis to the thigh, in a man-
ner analogous to the ropes which support the
mast of a ship in its position, but of course ca-
pable of variation in their respective lengths,
according to the necessities of varying move-
ments of the joint.
In certain cases an argument of the existence
of a provision may sometimes be drawn from
the necessity of the provision; but in this case
probably an argument so raised would be
scarcely admissible, because the economy of
the articulation has sometimes no such provi-
sion as that referred to, and consequently its
absence neutralizes altogether the argument
drawn from its necessity. Yet, when the
enormous muscles which surround the joint
are taken into consideration—muscles, to
which there are none in the body approaching
in power—we cannot avoid the conclusion that
their tendency must be to bring the opposing
articular surfaces into forcible contact with
each other; and that by causing a considera-
ble amount of friction, some impediment to
motion, and a still more important consequence,
the uzure and wearing away of the cartilages,
would be induced. The interposition of a pro-
vision which obviates these consequences, if
not absolutely necessary, has a very near claim
to be considered as necessary; and we are al-
most justified in its assumption as an argu-
ment. In Dr. Knox’s memoir there is a refer-
ence made to cases in which the round liga-
ment was wanting. These cases, in my opin-
ion, afford corroborative evidence of the uses
which are here assigned to it. He states, that
in a joint where there was observed such deli-
ciency, “ atrophy or uzure of the cartilages had
commenced, and made considerable progress,
as well on the head of the femur as on the cor-
responding acetabular surface.” At another
part of his memoir he says, “ 1 have been in-
formed by my friend Mr. Dick, of the Veteri-
nary College, that it is not very unusual to
find the round ligament of the femur absent in
the horse.” In these cases he found “atrophy
of the cartilages of incrustation, and the con-
version of a part, at least, of the abraded sur-
faces into the ivory structure.” Although
some doubt is expressed as to which of the
conditions was primary and which secondary,
Mr. Dick is of opinion that the division of the
licrament had precedence, and was produced by
accident. On the supposition of the correct-
ness of which opinion, the cause of the other
changes described admit of satisfactory expla-
nation, viz., that they were induced by fric-
tion and uzure of the surfaces, from an absence
of the countervailing provision ; while the fore-
going experiments sufficiently show, that the
adequate countervailing provision against fric-
tion and uzure of surface exists in the hip so
lono as the round ligament remains entire.
Independently of the beauty of the arrange-
ments of this ligament, considered as a mecha-
nical contrivance, there occur to me considera-
tions in reference to it, in connection with dis-
eased actions attacking the joint, which merit
some attention, and which give to it considera-
ble importance in the practical investigations
undertaken with the view to a correct diag-
nosis.
It is well known to the profession, thatin his
admirable work on Diseases of the Joints, Sir
B. Brodie has recommended the surgeon, in
the examination of the hip-joint, when affected
with disease, to apply his hand to the heel of
the patient, so as to press the head of the fe-
mur against the acetabulum; and certain in-
ferences are to be drawn from the presence or
absence of pain under the operation. I bus it
is said that in hips affected with ulceration of
their cartilages, violent pain is the consequence
of such pressure: but, on the contrary, in the
inflammation of the synovial membrane, no ag-
gravation of pain is produced ; and the diag-
nosis of one disease from another is made ac-
cordingly. The general accuracy of Sir B.
Brodie’s observations would be a sufficient
guarantee for the correctness of this rule for
forming a diagnosis, were it not confirmed by
the observation of others and of my own. Yet,
notwithstanding the correctness of the rule,
from an examination of the attachments and
uses of the round ligament, it appears to admit
of some doubt, whether the rule is not falla-
cious in certain cases. Thus, supposing a
disease to have its origin in the synovial appa-
ratus surrounding that ligament, provided its
tension were increased, we should expect a
corresponding increase of pain. Pressure with
the hand on the heel produces this increase of
tension; and if with it there be such corres-
ponding increase of pain, we are taught, by the
general application of the rule, to conclude that
the cartilages are ulcerated, and that, only se-
condarily, probably the ligaments themselves
may become diseased. Again, the absence of
pain, when the heel is pressed, is supposed to
denote absence of ulceration of the cartilages,
and inferenlially that the ligaments are the
seat of disease : a conclusion which may be
generally correct, but which may admit of ex-
ceptions, from the limitation of forcible contact
between the opposing articular surfaces, in-
duced by the uses to which the round ligament
is destined (as stated above) in the economy
of the articulation. According to this view,
neither the presence nor absence of pain on
pressure of the heel are, in every case, to be
considered as unequivocal evidence of the pre-
sence or absence of ulceration of the cartilages,
nor of the absence or presence of incipient dis-
ease of the synovial apparatus.—Ibid.
On Dislocations of the Sternal Extremity of
the Clavicle backwards. By M. V. Morel.—
The author has had three opportunities of exa-
mining this rare accident at La Charite and La
Pitie, and from a comparison of these cases
with the only three others published, has
drawn up an essay, of which the following is
an abstract:
The causes of this dislocation are forces act-
ing indirectly by pushing the shoulders violent-
ly forwards, or by suddenly pulling the arm
forward while the trunk is fixed, or directly by
sudden blows on the front of the clavicle. The
latter cause is by far the most rare.
The symptoms at the moment of the acci-
dent are extreme pain at the base of the neck,
with syncope, or a tendency to it, and gene-
rally more or less difficulty of breathing. The
position of the limb is nearly the same as in
fractures of the clavicle.
When the dislocated bone has passed back-
wards and downwards, its scapular extremity
is always more than naturally prominent, and
the clavicular end is lost sight of; but, if the
patient be thin, and the parts not swollen, it
can be felt pushed in behind the sternum. A
depression more or less painful to the touch,
indicates the vacancy at the sternal fossa, and
the external portion of the sterno-mastoid mus-
cle is obviously turned backwards and inwards;
but none of these last signs can be discerned
if the patient be fat, or if the injured parts be
much swollen. The shoulder does not readily
move; but if the whole strength of two men be
applied to pull it backwardsand outwards, the
parts regain their natural relations, and the
luxation is reduced; but the instant the exten-
sion is remitted, a rubbing sound, and the re-
appearance of the deformity, announce the re-
production of the displacement.
The dislocation backwards and downwards
is very often, by the efforts at reduction or
other forces, converted into one backwards hnd
upwards. In this case, the end of the clavicle,
instead of being fixed, is moveable, and, pro-
jecting inwards and upwards, forms above the
sternum a small, hard, round tumour, which
moves with every motion of the shoulder, and
may be easily reduced and pushed out of the
joint again.
The reduction of the dislocation backwards
and upwards then is perfectly easy; that of the
dislocation backwards and downwards is best
effected by pulling the upper part of the arm
horizontally backwards and outwards, while
the elbow is held against the side of the chest.
But the main difficulty is to keep the head of
the bone, when reduced, in its place; and this
may be best accomplished by M. Velpeau’s
bandage, which fixes the arm with its elbow
on the front of the chest, and the hand on the
opposite shoulder, and is itself retained in
place by a second bandage similarly applied
and thoroughly starched. It must be worn for
forty or fifty days.—Jinn, de la Chirurg.
Case of Tumour of the brain, with Hydroce-
phalus, and arrest of Development of the Uterine
System. By Dr. O’Bryen.—Mary Morgan,
aged 20, when about two years of age, had
an acute attack of hydrocephalus, which ap-
peared to continue in a chronic form during the
rest of her life. At the age of seven, during
another attack, she lost the sight of her left
eye. When about fourteen years of age, the
fits to which she was subject became more se-
vere, and of longer duration ; and when about
eighteen, after feverish symptoms, and an
epileptic fit, which lasted two hours, with
slight delirium, the right side became para-
lysed. About four months before her death she
frequently slept for a whole week, during
which she suffered from convulsions every
four ’or five hours. The power of speech,
which had been gradually becoming more im-
perfect, was lost for some days before her
death, as was also the power of swallowing ;
she also partially lost the use of the right eye.
She died when about twenty years of age. The
expression of her countenance was very child-
ish. Menstruation never appeared; nor did
any change take place in her manners or
habits, as is usual at this period of female life.
She showed no preference for the society of
the male sex, but rather shunned them, pre-
fering the toys of childhood, and children of
five or six years old for her playmates.
The dura mater was thickened, white, and
extremely resistant. The arachnoid was
thickened, and of a milky whiteness. The
convolutions of the brain were almost oblite-
rated, and the substance softened. After re-
moving a slice of about half an inch from the
hemispheres, the lining membranes of the
lateral ventricles were brought into view.
They were white, tense, very resistant, and
distended with serous fluid. When the se-
rous fluid, which was about eighteen ounces
in quantity, had escaped, the brain lost its
round appearance, and became flattened. When
the ventricles were laid open, a tumour was
found arising from the anterior part of the left
optic thalamus covered by the lining membrane
of the ventricles, which it had pushed before it.
When the brain was removed, a second branch
of the same tumour was found extending along
the petrous portion of the temporal bone, pe-
netrating the tentorium cerebelli above the in-
ternal auditory foramen, and adhering firmly
to the arachnoid covering the left lobe of the
cerebellum. The tumour was composed of
calcareous matter; and measured from its base
upwards of two inches, and from its base to
the end of its lateral branch, two inches and a
quarter, varying from half to one inch in
breadth. Near the base of the tumour was a
notch, under which the third, fourth, and sixth
cerebral nerves, with the first branch of the
fifth pair passed. The base of the brain pre-
sented a loss of substance corresponding to the
size of the tumour, commencing to the right of
the tuber annulare, wffiich was sound, includ-
ing a fourth of an inch of the crus cerebri, two
convolutions of the middle lobe, to the depth
of nearly one inch, on the outside leaving the
fifth pair of nerves, and on the inside the cor-
pora albicantia untouched. The tumour then
penetrated the left optic thalamus, destroyed a
part of the corpus striatum, the left optic nerve
posterior to its commissure, and the first pair
of nerves at their origin. The pineal gland
could not be found. On inspecting the base
of the cranium, the left anterior and posterior
clinoid processes were found absorbed ; and
the tumour, with a narrow7 base, w7as found ad-
herent to that portion of the membrane lining
the dura mater, which covers the left carotid
artery after it enters the cranium.
The cerebellum was the only part of the ce-
rebral mass which appeared to be perfectly
healthy, if the adhesion of its membranes to
the tumour be excepted.
Nothing unusual was met with in the tho-
racic and abdominal viscera, with the excep-
tion of the left lobe of the liver, which was
nearly as large as the right. The uterus ex-
isted only in miniature; it measured only one
inch in length, one line and a halfin thickness,
and four lines in breadth. The ovaries were
each about the size of an almond, and when
divided presented one or two cavities, 'rhe
Fallopian tubes were well formed, but their
openings into the uterus were much larger than
usual. The os tincae were about the size of a
small pea, and very soft. The vagina was
small, and had very few rugae. No hair ex-
isted on the pubes, and the mammae were not
developed.
The above case appears to be worthy of no-
tice from two circumstances connected with it;
first, that the destruction of the left optic nerve
posterior to the commissure was followed by
loss of vision of the left eye, whilst the hu-
mours retained their transparency; and secondly,
that though the generative organs presented
not the smallest developement, the cerebellum
was not involved in the disease, but appeared
perfectly healthy,—Edinburgh Medical and
Surgical Journal, from Dublin Journal of Med.
Sciences, Jan. 1841.
We had occasion,when speaking of the opera-
tion of tenotomy in a former number, to.referto
a/jase of fracture of the olecranon processes,—
the one consequent to the other—and to de-
scribe the extent of shortening observable in
the extensor cubiti from gross error in the
treatment, together with the limitation of the
motions of the forearm resulting from the acci-
dent. In the following case we are presented
with two very similar accidents affecting the
tendons of both recti femores from muscu-
lar contraction alone. Both this observation
and our own very clearly show the fallacy of
supposing that the elongation of a contracted
muscle can be effected by dividing and then
mechanically extending its tendon without very
seriously diminishing its functional power,—a
fallacy most strangely advocated by many of
the defenders of indiscriminate tenotomists
and myotomists on club-foot and deformities
of the spine.	R. C.
Rupture of the Rectus Femoris of both Thighs.
By William England, M. D.—Mr. Gran-
tham’s case of rupture of the rectus femoris
being of considerable interest, I beg leave to
record one which has occurred in my practice,
and which I think you will find equally so, be-
ing a double rupture of the recti muscles of
the thigh; and so far analogous to the one re-
lated by that gentleman, in proving the possi-
bility of the occurrence of “ rupture of the
tendon of the rectus femoris muscle, unattend-
ed with fracture, dislocation, or laceration of
the adjacent parts.”
In the summer of 1838, a gentleman of ro-
bust appearance for his age, 73, consulted me
for chronic dyspepsia, accompanied with se-
vere cardialgia. Two years previously he
had an attack of paralysis, which left the sen-
sibility of the left arm much impaired. He
had no power of locomotion except on crutches.
There was complete inability of extending the
lotver extremities, owing to the rupture of the
rectus femoris of each thigh ; a groove being
left above each patella as deep as the breadth
of a finger.
In the following autumn, Mr.---------had a
severe attack of the erysipelas of the face, ex-
tending over the scalp. From this attack he
recovered with great difficulty; and having
visited him with Mr. Culledge, of March, his
medical attendant during a long series of years,
I am enabled to give the report of that gentle-
man, communicated to me. About fourteen
years ago, when in the act of running, Mr.
------fell down (forwards,) and, on attempt-
ing to rise, found that he had injured the
knee. Mr. Culledge was immediately called
in, and found the tendon of the rectus femoris
of the right thigh completely ruptured. Under
the hope that re-union might be effected, he
was placed in as favourable a position as
could be for that purpose ; but he would not
allow it to remain so for one hour, nor could
he be prevailed upon to do so. About four
years after the above accident, while walking
in his stack-yard, Mr. ---’s left foot came
suddenly in contact with a piece of wood, and
he again fell forward, and with precisely the
same consequences to the left limb. From the
first accident he was compelled to use crutches;
and although he posseesed the power of loco-
motion to a limited degree, yet, upon the
slightest flexure of either knee, he would at
any time have fallen down without such sup-
port. No injury was done to the adjacent
parts at either period.—London Med. Gaz.
Interments in the City and Liberties of Phi-
ladelphia, from the iPith of November to the
4th of December.
>	>	n
ex	ex	tr
Diseases. £. e Diseases.	e
“	d	S®	nJ
P	?
Asphyxia,	0 2 Brought for ward,HQ 36
Apoplexy	1 0 Inflammation of
Cancer of breast, 1	0 the Peritonaeum,1	0
Croup,	0 1 Injury of the
Congestion of	Spine,	1	1
brain,	1	1	Marasmus,	0	1
Colic,	0	1	Old age,	1	0
Consumption of	Palsy,	1	0
the lungs,	18	2	Pleurisy,	1	0
Convulsions,	0	5	Small pox,	0	6
Dropsy,	0	1	Still-born,	0	7
--------Abdominal, 10	-----
	Head,	0 3 Total,	92—41 51
-------------------------------------------------------Breast,	0 1
Disease of the	-----
kidneys,	1	0
----Lungs, 0	1 Of the above, there
Drowned,	1	0	were	under 1 year,	35
Dysentery,	1	1	From	1	to	2	6
Debility	0	3	2	to	5	4
Fever, Typhus, 12	5 to 10	1
----Typhoid, 2 0	10 to 15	0
------------------------------------------Scarlet,	0 2	15 to 20	5
Gangrene of	20 to 30	14
lungs,	1	0	30	to	40	7
Haemorrhage,	0	1	40	to	50	5
Inflammation of	50 to 60	7
the Bronchi,	0	3	60	to	70	4
----Lungs,	3 5	70 to 80	3
	Liver,	3 1	80 to 90	1
------------------------------------------ Larynx 0	1		
----Total,	92
Carried forward, HQ 36i
				

